# Classification of So-Called Non-Covalent Interactions Based on VSEPR Model

**DOI:** 10.3390/molecules26164939

**Published:** 2021-08-15

**Authors:** Sławomir J. Grabowski

**Affiliations:** 1 Polimero eta Material Aurreratuak: Fisika, Kimika eta Teknologia, Kimika Fakultatea, Euskal Herriko Unibertsitatea UPV/EHU & Donostia International Physics Center (DIPC) PK 1072, 20080 Donostia, Spain; s.grabowski@ikerbasque.org; 2 Ikerbasque, Basque Foundation for Science, 48011 Bilbao, Spain

**Keywords:** valence-shell electron-pair repulsion model, hydrogen bond, σ-hole bond, π-hole bond, electron charge shift

## Abstract

The variety of interactions have been analyzed in numerous studies. They are often compared with the hydrogen bond that is crucial in numerous chemical and biological processes. One can mention such interactions as the halogen bond, pnicogen bond, and others that may be classified as σ-hole bonds. However, not only σ-holes may act as Lewis acid centers. Numerous species are characterized by the occurrence of π-holes, which also may play a role of the electron acceptor. The situation is complicated since numerous interactions, such as the pnicogen bond or the chalcogen bond, for example, may be classified as a σ-hole bond or π-hole bond; it ultimately depends on the configuration at the Lewis acid centre. The disadvantage of classifications of interactions is also connected with their names, derived from the names of groups such as halogen and tetrel bonds or from single elements such as hydrogen and carbon bonds. The chaos is aggravated by the properties of elements. For example, a hydrogen atom can act as the Lewis acid or as the Lewis base site if it is positively or negatively charged, respectively. Hence names of the corresponding interactions occur in literature, namely hydrogen bonds and hydride bonds. There are other numerous disadvantages connected with classifications and names of interactions; these are discussed in this study. Several studies show that the majority of interactions are ruled by the same mechanisms related to the electron charge shifts, and that the occurrence of numerous interactions leads to specific changes in geometries of interacting species. These changes follow the rules of the valence-shell electron-pair repulsion model (VSEPR). That is why the simple classification of interactions based on VSEPR is proposed here. This classification is still open since numerous processes and interactions not discussed in this study may be included within it.

## 1. Introduction

Numerous interactions have been analysed in various studies [1,2,3,4,5,6,7] since they are often preliminary stages for chemical reactions and processes [8,9,10,11,12]. One can mention the proton transfer in hydrogen bonded systems [13,14,15,16,17,18], the interrelations between interactions and catalysis [19,20], the arrangement of molecules and/or ions in crystal structures and, in general, crystal engineering [5,6,8]. Other issues may be noted. For example, the hydrogen storage [21,22,23] and the storage of other compounds that are crucial in such processes as distillation, crystallisation, hydrolysis, and numerous less or more complex operations related to the technology and industry but particularly related to variety of interactions. This is why intra- and intermolecular interactions are the subject of various studies; the understanding of their nature and mechanisms deepens our understanding of the above-mentioned reactions and processes.

The name “noncovalent interactions” (NCIs) is often applied in various studies in spite of the fact that it does not match the properties of such interactions [24]. This is due to the fact that the term covalency is related to electron charge shifts, and such shifts are more pronounced if stronger interactions occur [25]. Hence, it is strange to name interactions as noncovalent if for the majority of them the pronounced electron charge shifts are observed. These shifts are also related to the interaction energy terms such as the polarization and the charge transfer [25]. Other names of stabilizing terms related to electron charge shifts are used in various studies [26]. However, these names and their physical meanings depend on the decomposition scheme applied for the division of the total interaction energy; different nomenclatures are used in different decompositions [26]. The electrostatic and dispersion contributions occur often as other attractive terms in different decomposition schemes. The Pauli repulsion term occurs most often in such schemes. 

This is why it is better to apply other names for so-called noncovalent interactions. One can mention the “secondary bond” (or bonding) term that is often applied for interactions that are not typical covalent bonds. This term was introduced by Alcock to describe atom-atom intra- and inter-molecular contacts in crystals that are longer than typical bonds and shorter than the corresponding sum of their van der Waals radii [27]. The description of Crabtree reflects the meaning and the range of this term adequately: “Secondary bonding is best thought of as incipient hypervalency and occurs when a main group element with an octet structure, EX_x_, interacts with the lone pairs of one or more neighbouring groups, Y, that bind weakly to E to give rise to E∙∙∙Y distances of intermediate length, lying between full bonds and mere contacts” [28].

The interrelation between the hypervalency and secondary bond is also discussed later here. The simple example may be presented that concerns the hydrogen bond. The A-H∙∙∙B designation is applied in various studies for hydrogen bond bridges. A-H is a proton donating bond, that may be named as primary bonding (covalent bond more or less polarized) and B is the proton acceptor belonging to the electron donating unit. The H∙∙∙B link that is a weaker interaction than the A-H bond is named as the secondary bonding. However, if this link is strong (it is sometimes extremely strong as in the [FHF]^−^ anion [29,30,31]), the hydrogen atom situated between two other centers may be considered as hypervalent [28]. 

Other names may be used to describe so-called noncovalent interactions. The name Lewis acid–Lewis base interactions may be applied [24]. The process of the electron charge shift related to the covalency is well determined for the latter term since this shift from the Lewis base unit to the Lewis acid unit is observed. For example, from the unit containing the B-centre to the unit with the A-H bond in the A-H∙∙∙B hydrogen bonded complexes. Very often, in local interactions, contacts between centres of opposite charges are observed, as in the H∙∙∙B contact of the hydrogen bond, for example. However, there are cases where centres of the same charge sign are in a contact, as for the C-Cl∙∙∙B halogen bonds with the negatively charged Cl-atoms. It was pointed out, however, that the electrostatic potentials of surfaces being in a contact are more proper to describe stabilizing interactions [32,33,34]. For various A-X (X is a halogen atom) bonds the positive electrostatic potential is observed in the elongation of this bond thus the halogen centre may be linked with the electron donor. Very recently the term “counter-intuitive” was introduced to describe interactions that correspond to contacts between regions characterized by the same sign electrostatic potentials [35]. There are examples of stabilizing counter-intuitive halogen bonds since not only the electrostatic term of energy of interaction but also the polarization term should be taken into account [35]. There are also weakly bonded by dispersion forces species, as for example methane molecules, or noble atoms. Such systems are not classified as linked by the Lewis acid–Lewis base interactions, but rather as the van der Waals complexes [24].

## 2. The σ-hole Bond and π-hole Bond Concept and the Classification of Interactions

The hydrogen bond is an interaction that was analysed in various studies due to its crucial role in numerous processes, including life processes [36,37,38]. There are different types of the hydrogen bond as the A-H∙∙∙B three-centre-four-electron (3c-4e) ones or the A-H∙∙∙π and A-H∙∙∙σ hydrogen bonds where π-electrons and σ-electrons, respectively, play a role of the proton acceptor [38]. These classes of the hydrogen bond correspond to the type of the electron donor. However other classifications are also known. 

The lithium bond, A-Li∙∙∙B [39,40,41], is the next interaction that was announced and described in early studies. One could expect very similar properties of the lithium bond to properties of the hydrogen bond since the only difference between these interactions is that in the former one the lithium is located between A and B centres instead of the hydrogen. However, these interactions differ significantly between themselves [41].

**Scheme 1:**
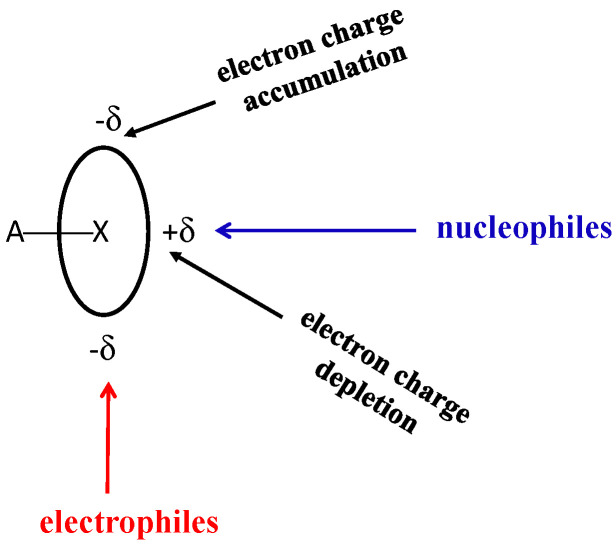
The dual role of halogen as the Lewis acid and Lewis base centre.

It was discussed in numerous studies that another interaction, the halogen bond, A-X∙∙∙B (X marks the halogen centre), may be treated as the counterpart of the hydrogen bond [42,43]. This may be surprising since the electronegative halogen, X, plays a role of the Lewis acid centre here. The Lewis acid properties of halogen centres were a subject of discussions and polemics. The dual character of such centres was explained by the anisotropy of electron charge distributions around halogen nuclei, which results in the Lewis acid properties approximately in the elongation of A-X bond and the Lewis base properties in the perpendicular direction, or nearly so [44] (see Scheme 1). 

The other explanation of the unique properties of halogens is based on the σ-hole concept [45,46,47]. According to this concept, the depletion of the electron charge in the elongation of the A-X bond results in the increase of electrostatic potential in this region, up to positive values. This region is named as the σ-hole. The latter depletion of the electron charge at the halogen is connected with the inclusion of this electron charge in the A-X sigma bond. Consequently, the excess of the electron charge in the perpendicular direction and near to this direction also occurs. It is worth to note here that not only the depletion of the electron charge in elongation of bonds is responsible for the positive electrostatic potential at the Lewis acid centre; the important contribution of the nucleus cannot be ignored [48]. 

**Figure 1:**
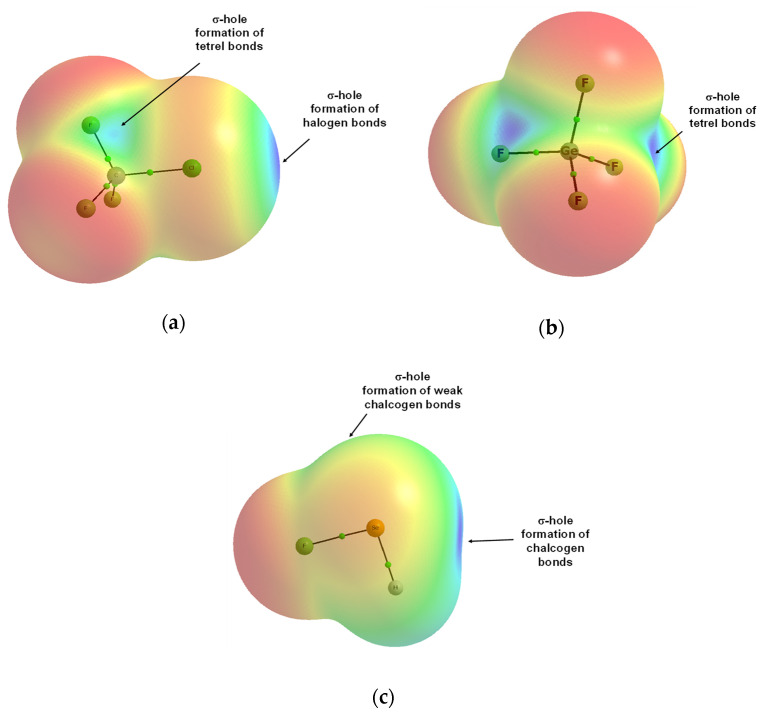
The electrostatic potentials for the 0.001 au surfaces of the electron density for the F_3_CCl (**a**), GeF_4_ (**b**) and SeFH (**c**) species; from the maximum positive EP (blue) to the minimum negative EP (red). The EPs correspond to MP2/aug-cc-pVTZ results.

A similar situation to this one described for halogens occurs for other elements of main groups. In the elongation of bonds to the elements of 16, 15, and 14 groups, the regions of the positive EP are observed that often results in interactions of these elements with the electron donors [32,33]. The corresponding interactions are named as the chalcogen [49,50,51,52,53,54], pnicogen [55,56,57,58,59], and tetrel bonds [60,61,62,63,64], respectively. Even for noble gas elements such positive EP regions are observed that may be linked with the Lewis base units through aerogen bonds [65]. Figure 1 presents computed electrostatic potentials, EPs, on the 0.001 au surfaces of the electron density for the F_3_CCl, GeF_4,_ and SeFH species.

The region of the positive EP in the elongation of the C-Cl bond for the F_3_CCl moiety is observed. One can also see here the regions of positive EP in elongations of F-C bonds at the carbon centre. The slight depletion of the electron charge at C-centre in the extension of the Cl-C bond is not sufficient for the increase of EP up to the positive value. The Cl-region of positive EP leads to the formation of halogen bonds while the occurrence of σ-holes at the carbon centre may lead to the formation of tetrel bonds. A similar situation occurs for the GeF_4_ species where four σ-holes at the carbon in elongation of F-Ge bonds may form tetrel bonds with electron donors. The positive regions of EP at F-centres are not observed for the F_3_CCl and GeF_4_ species. In the case of SeFH molecule the chalcogen bonds may be formed through σ-holes at the selenium centre, in elongation of the F-Se and H-Se bonds. The former σ-hole is characterized by greater positive EP value than the latter one.

There are several findings concerning σ-holes and electrostatic potentials that are not a subject of this study, however. One can mention that usually for the same group elements acting as the Lewis acid centres, the heavier ones possess stronger electron accepting properties [32,33]. Thus, for example, for halogen bonds, the C-F∙∙∙B interactions are not usually energetically stable, the C-Cl∙∙∙B ones are stable but rather weak, and the strength of C-Br∙∙∙B halogen bonds is comparable to the corresponding hydrogen bonds while the C-I∙∙∙B are often stronger than hydrogen bonds. This is an approximate trend that changes depending on environment, the kind of the electron donor centre (B), etc. For example, the Lewis acid properties of fluorine centres were analyzed for various species in crystals and in a gas phase where fluorine can possess positive electrostatic potential area if it is linked with strongly electron-withdrawing residues [66]. The similar trends of the increase of positive EP at the heavier Lewis acid centre are observed for other groups of elements. It is also observed that the substituent may enhance the σ-hole, and consequently may increase the positive EP at this area [32,33,66]. The SeFH species are presented in Figure 1c. It is used as an example since the increase of EP at selenium centre is observed due to the electron withdrawing properties of fluorine. The other σ-hole at Se centre, in the extension of the H-Se bond, is characterized by a lower EP value.

There are other regions of positive EPs that may act as the electron acceptors. These are π-holes observed for some planar molecules or for planar fragments of molecules [32,33,48]. Such regions often occur at elements of 13th group (triel elements). For example, boron forms three B-H or B-X covalent bonds by using sp^2^ hybrid orbitals in boron trihydride, BH_3_, and boron trihalides, BX_3_, respectively, that results in a planar trigonal molecular system [67,68,69,70,71]. Such compounds are used as Lewis acids in the syntheses since the boron center reveals strong affinity to electrons. These Lewis acid properties of boron are connected with its electron structure since boron is electron deficient in the above-mentioned compounds; it has six electrons in the outer shell. The interaction with electron donating ligands leads to the complement to the electron octet and the tetrahedral boron structure [12]. 

**Figure 2:**
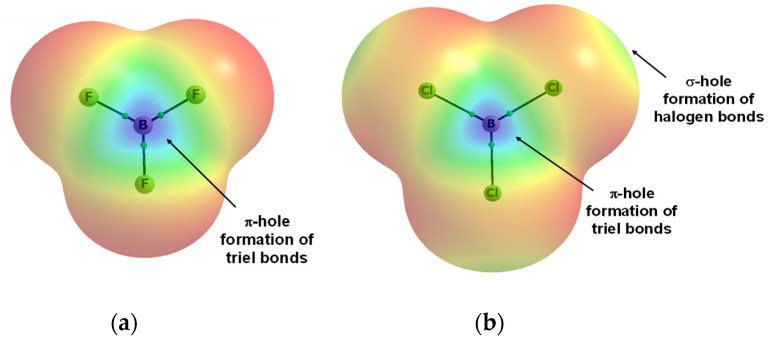
The electrostatic potentials for the 0.001 au surfaces of the electron density for the BF_3_ (**a**) and BCl_3_ (**b**) species; from the maximum positive EP (blue) to the minimum negative EP (red).

Figure 2 shows EP surfaces characterized by the electron density of 0.001 au for the BF_3_ and BCl_3_ molecules. For both species, the π-hole possessing positive EP at boron centre occurs. The surfaces at BF_3_ fluorine atoms are characterised by negative EP while for the BCl_3_ molecule, the σ-holes are observed at chlorine centres in elongations of the B-Cl bonds. In such a way the BCl_3_ species may interact with electron donors forming triel bond (through boron) and halogen bonds (through chlorines).

It is worth mentioning that the π-hole may be associated with a specific atom or with two or more atoms. For example, in benzene and its derivatives π-holes are craters above and below the rings [48]. The origin of holes related to the depletion of the electron charge was analysed in view of molecular orbitals. Three types of interactions were analyzed in this approach: σ-hole, π-hole and δ-hole bonds [72]. However, more popular are the names of interactions corresponding to the groups of periodic system. The tetrel (14 group), pnicogen (15 group), chalcogen (16 group), and halogen bonds (17 group) were analysed early in terms of the σ-hole concept by Politzer, Murray, Clark and coworkers [32,33,34,45,46,47]. The tetrel bond was analyzed in detail by Politzer and coworkers [61] but this name was introduced in later studies [62,63]. The remaining, mentioned above, names of interactions were in use in earlier studies, before introduction of the σ-hole concept. In later studies other names related to interactions corresponding to main groups elements were introduced; these are: the aerogen bond (18 group) [65], triel bond (13 group) [73,74], alkaline earth bonds (2 group) [75], alkali bonds (1 group, excluding hydrogen) and interactions of the transition metals, regium bonds (10 and 11 groups), and spodium bonds (12 group) [76]. The discussion on interactions related to groups of the periodic system was performed in a recent study [76]. It is worth mentioning that the names related to single elements appear also from time to time in various studies. For example, the hydrogen bond [36,37,38], the lithium [39,40,41], carbon [77], fluorine [78], gold [79,80,81], beryllium [82,83,84], and magnesium [75,85,86,87,88] bonds often occur. One can see that these names, both related to groups and to single elements, are connected with the Lewis acid properties of the centre involved in the interaction, which was discussed in one of the latest studies [25]. However, names related to the Lewis base properties of the centre being in a contact with the electron acceptor occur sometimes; one can mention hydride and halide bonds [25,89].

There are other examples of interactions, such as dihydrogen [90,91,92,93,94,95,96,97] and dihalogen [98,99] bonds, the previous one of which concerns contact between two H-atoms possessing opposite charges. In the case of dihalogen bonds, at least two sub-classes should be mentioned. There are other interactions that are hardly fitted into any classification. Of these, anion-π and stacking interactions may be mentioned [5,6].

One can see that the nomenclature used in various studies is not uniform. There are numerous reservations to the previously used names that are presented below, some of which were mentioned before here.

In general, the classification of interactions is not systematic and uniform.

The majority of names of interactions refers to the Lewis acid centres, less often some names concern the Lewis base centres.

Some of names concern groups, and some of them only single elements.

The properties of interactions within the same group of the periodic system may differ significantly. For example, halogen bond with the chlorine centre possesses different properties than such interaction with the iodine centre.

There are numerous sub-classes within any interaction considered, for example, there are numerous sub-classes of the hydrogen bond or even of dihalogen bond.

In view of these reservations the following question arises: did it make sense to introduce new names for interactions that were analysed in recent and much earlier studies? It is discussed in the next sections of this article.

It is worth to refer here to the recent study of Politzer, Murray, and Clark [48] where the following though about names of interactions is presented: “In recent years, there has developed a tendency to label σ- and π-hole interactions in accordance with the column of the Periodic Table in which the σ- or π-hole atom appears. (…) This may have some advantages, but it obscures the key unifying facts that σ-hole interactions are one fundamentally similar group, occurring along the extensions of covalent bonds or parallel to them, the π-hole interactions are a second fundamentally similar group, perpendicular to planar portions of molecules. The basic differences are not within each group but rather between the groups, in directionality”.

## 3. The Electron Charge Shifts Accompanying Lewis Acid–Lewis Base Interactions

The formation of hydrogen bond and of other Lewis acid—Lewis base interactions (secondary bonds) leads to numerous energetic, structural, and electronic changes in the linked units. Various phenomena that accompany the formation of complexes have been discussed in recent reviews [24,25,28]. It has been pointed out that two main forces steer such secondary bonds. This is the electrostatic component related to the σ-hole concept where electrostatic potentials of molecular spheres determine the arrangement of interacting units and the covalent character that is often described by the n → σ* orbitals’ overlaps [28]. For example, for the A-H∙∙∙B hydrogen bond, the n_B_ → σ_A-H_* overlap is often treated as a signature of the occurrence of this type of interaction [100,101]. n_B_ marks lone electron pair of the proton acceptor centre B in the Lewis base unit while σ_A-H_* is the antibonding orbital of the A-H bond belonging to the Lewis acid unit. The covalent part of interaction is strictly related to the hypervalency phenomenon. If we limit to the elements of main groups as centres, then “a hypervalent molecule is one that has more than four pairs of valence electrons around the central atom” [28]. These electron pairs are understood here as covalent bonds to this centre and as nonbonding pairs such as lone electron pairs. This is a similar categorization of electron pairs as in the Valence-shell Electron-pair Repulsion model, VSEPR [102]. 

In the case of the central atom that obeys the octet rule, the additional interaction (secondary bond) may lead to the increase of the electron charge at this centre and thus to the hypervalency [25]. This is why the secondary bonds, especially the strong ones, are equated with the hypervalent bonds where the interacting centre does not obey the octet rule. One may refer to the 4e-3c structure (four electrons—three centres), marked as A-Z∙∙∙B where the Z-centre involved in strong interaction with B that donates the lone electron pair is hypervalent in the case of strong interactions [25,28]. The 4e-3c structure may be related to numerous interactions analysed in various studies, to hydrogen bonds, halogen and pnicogen bonds, and many others. The [FHF]^−^ anion is an example of a very strong hydrogen bond [29,30,31] where the H-centre may be considered as the hypervalent one [28]. Hoffmann has pointed out that the 4e-3c bond, and the n → σ* overlap described earlier here, are alternative ways to describe the same phenomenon [103]. It is worth noting that for systems of three atoms such as those discussed here the term of 3c/4e hypervalent ω-bond was introduced [101] (3c/4e designation follows ref [101]). 

The hypervalency term is discussed and questioned in some studies [104,105]. It was pointed out, for example, that the analysis of electron charges of numerous centres that are usually indicated as the hypervalent ones, shows that the number of electrons attributed to valence shell often only slightly exceeds, if they do at all, number eight [104,105]. Additionally, it was justified in one of recent studies that there are mechanisms and processes that try to protect the octet or doublet structure of the central atom if this is the main group element or particularly H-atom, respectively [25]. These mechanisms are described below.

**Scheme 2:**
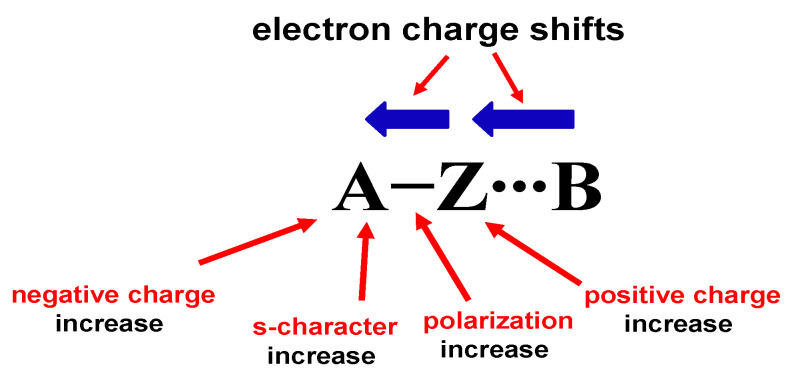
The formation of hydrogen bond, halogen bond, and other interactions.

Scheme 2 presents main processes that accompany the formation of hydrogen (Z = H) or halogen (Z = X) bond [106]. It is also approximately in force for other σ-hole and π-hole interactions [12,24,25,74]. The Z centre that obeys the octet or doublet rule if interacts with any electron donor ligand may contain excess of the electron charge since the electron charge transfer from the B unit into the A-Z one is the main characteristic of the Lewis acid—Lewis base interactions. This could lead to hypervalency, but other phenomena also occur [25]. There is the further electron charge transfer from the Z-centre to the remaining part of the unit, mainly to the A-centre [25,100,101]. Thus, the increase of the positive charge of Z-centre is usually observed. These processes of the electron charge shifts were analysed for the halogen and hydrogen bonds in detail. Two other effects are observed as results of the formation of interaction, the increase of the polarization of A-Z bond and the increase of the s-character of the A orbital of A-Z bond. The interrelations between the s-character and other parameters for various hydrogen bonds were analysed in detail by Alabugin and coworkers [107] and further this analysis was performed for other interactions [108].

It is worth mentioning that these changes (Scheme 2) resulting from the formation of A-Z∙∙∙B secondary interaction may be also treated as a response to the Pauli repulsion which increases with the shortening of the Z∙∙∙B distance [25]. One may refer to one of decomposition schemes of the energy of interaction [109,110].

*ΔE_int_* = *ΔE_elstat_* + *ΔE_Pauli_* + *ΔE_orb_* + *ΔE_disp_* (1)

The term *ΔE_elstat_* corresponds to the quasi-classical electrostatic interaction between the unperturbed charge distributions of atoms. The Pauli repulsion, *ΔE_Pauli_*, is the energy change connected with the transformation from the superposition of the unperturbed electron densities of the isolated fragments to the wave function that obeys the Pauli principle through antisymmetrisation and renormalization of the product wave function. The orbital interaction, *ΔE_orb_*, corresponds to the electron charge shifts accompanying the complex formation. The attractive dispersion interaction energy term, *ΔE_disp_*, is also included in this decomposition (Equation (1)).

It was found for numerous types of interactions that the Pauli repulsion term correlates with the sum of the stabilizing interaction energy terms (orbital, electrostatic, and dispersion) [25,74]. However better correlation is observed between repulsion and orbital energy only. It means that mainly the attraction related to the electron charge shifts counteracts the repulsion. There are other examples of interrelation between the phenomena described above. However, it is worth mentioning that in the A-Z∙∙∙B interaction, the Pauli repulsion and electron charge shifts are interrelated with other processes that protect the octet or doublet electron structure of Z centre and of other centres of interacting units, particularly of the A-centre. The latter processes were analysed for the O-H∙∙∙O and C-H∙∙∙O hydrogen bonds since the calculations on complexes of water, H_2_O, hydronium cation, H_3_O^+^ and chloroform, CCl_3_H, were performed [111]. Excellent linear correlations were found between the polarizations of the proton donating bonds (C-H or O-H) and the mean polarizations of the remaining bonds of the Lewis acid unit; the increase of the former polarization correlates with the decrease of the latter mean value. It shows that various effects accompanying the hydrogen bond formation try to protect the doublet and octet structures of H-atom and of O or C centre, respectively.

## 4. Changes of Structures of Interacting Centres

The formation of intra- or intermolecular links leads to structural changes that mainly concern conformations of centres being in contact. These structural modifications are greater for stronger secondary bonds that are often classified as hypervalent bonds. Let us consider geometrical deformations concerning changes of conformations which accompany formation of tetrel bonds. In the case of tetrahedral conformation corresponding to the sp^3^ hybridization of tetrel centre (14 group element), there are four σ-holes in extensions of bonds to this centre [61,62,63]. The interaction of tetrel species through such σ-hole with the electron donating ligand known as the tetrel bond, may lead, in the case of strong interaction, to the trigonal bipyramid conformation or to the structure possessing geometry close to this conformation. It was discussed that the tetrel bond where the tetrel unit possesses the tetrahedral conformation may be treated as the preliminary stage of the S_N_2 reaction [63]. The transition state of this reaction corresponds to the hypervalent bond and has the conformation of a trigonal bipyramid [63].

**Figure 3:**
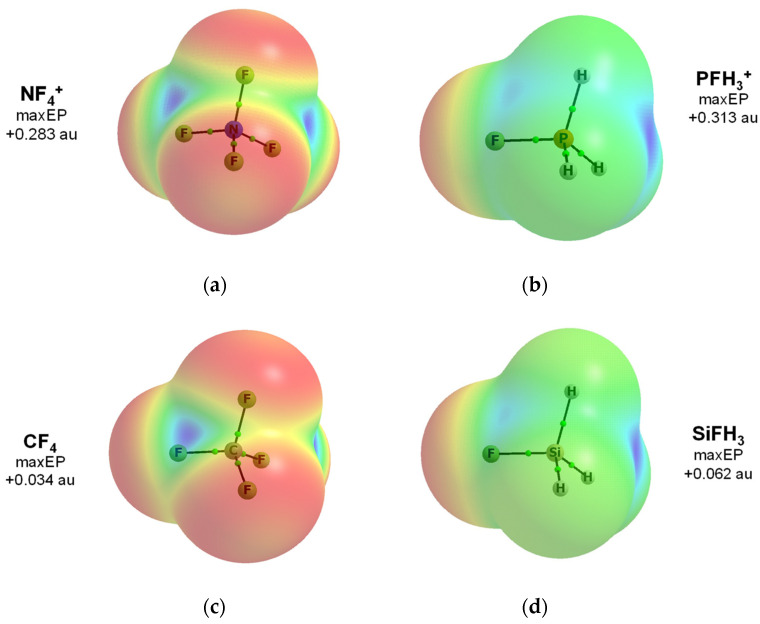
The electrostatic potentials for surfaces of the electron density of 0.001 au for NF_4_^+^ (**a**), PFH_3_^+^ (**b**), CF_4_ (**c**) and SiFH_3_ (**d**) species; from the maximum positive EP (blue) to the minimum negative EP (red); the EP values correspond to MP2/aug-cc-pVTZ results.

However, a similar situation occurs for the pnicogen (15 group element) cations characterized by the tetrahedral geometry which through σ-holes located at pnicogen centre may interact with electron donors that leads also to the trigonal bipyramid geometry [112]. Figure 3 presents two tetrel and two pnicogen species; the former tetrel species are neutral molecules while the later ones are cations, and all are characterized by tetrahedral structures. One can see that the greater values of electrostatic potential, EP, at the σ-holes are observed for pnicogen cations than for neutral tetrel species. For both types of interactions, for neutral tetrel bonds and for charge assisted pnicogen bonds, numerous correlations were found which show that for stronger interactions the systems are closer to the trigonal bipyramid structure. For very weak interactions the tetrahedral structure is only slightly disturbed. Hence one can see that both interactions, the charge assisted pnicogen bond and the tetrel bond, which concern elements of different groups possess very similar characteristics. This is another argument that current classifications of interactions are not the best ones.

The ammonia cation clusters, NH_4_^+^∙∙∙(H_2_)_n_ [113] and NH_4_^+^∙∙∙(NCH)_n_, [114] with n up to eight ligands, were analyzed. It was found that for the number of ligands less than or equal to four the N-H∙∙∙σ and N-H∙∙∙N hydrogen bonds are formed between ammonia cation and ligands. For more ligands than four the N∙∙∙σ (H-H bond) and N∙∙∙N σ-hole bonds (pnicogen bonds) are formed apart from the hydrogen bonds.

**Figure 4:**
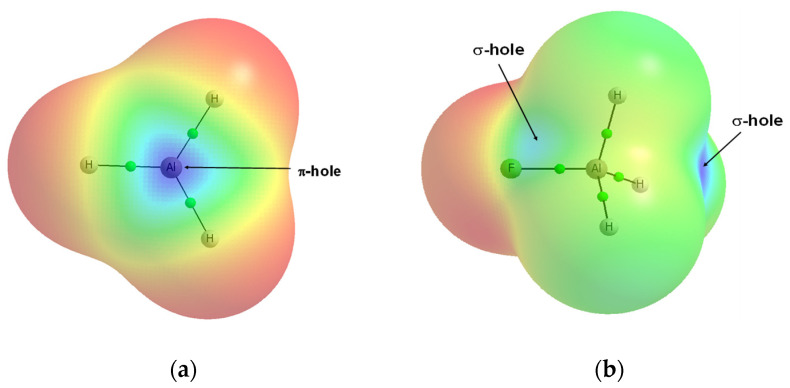
The EP surfaces for the electron density of 0.001 au for AlH_3_ (**a**) and AlFH_3_^−^ (**b**) species; from maximum positive EP (blue) to minimum negative EP (red).

Significant structural changes are also observed for the triel bonded systems [73,74]. The triel centres are often located in planar molecules or in planar fragments of molecules. Figure 4 presents the molecular graph of the aluminium trihydride, the surface of the electron density of 0.001 au is also presented with the map of the electrostatic potential, EP. One can see the EP maximum at the Al-centre that is attributed to the π-hole. This is the trigonal structure which is characteristic for numerous triel centres. A similar electron charge distribution that results in similar EP map is observed for boron trifluoride and boron trichloride (Figure 2). These triel centres may interact with electron donors that leads, in the case of strong interactions, to the change of the trigonal configuration of the triel centre into the tetrahedral structure [74].

Figure 4b presents the molecular graph of the AlFH_3_^−^ species that is a result of the H_3_Al∙∙∙F^−^ triel bond, with the surface corresponding to 0.001 au electron density. The EP map is presented (Figure 4b) with its maxium at the Al-centre. This EP maximum corresponds here to the σ-hole rather and not to the π-hole. The other σ-holes are observed in elongations of the H-Al bonds but they are characterized by lower positive EP values than the σ-hole located in the extension of the F-Al bond. One can see that the AlFH_3_^−^ species may be categorized as a slightly deformed tetrahedral structure.

**Figure 5:**
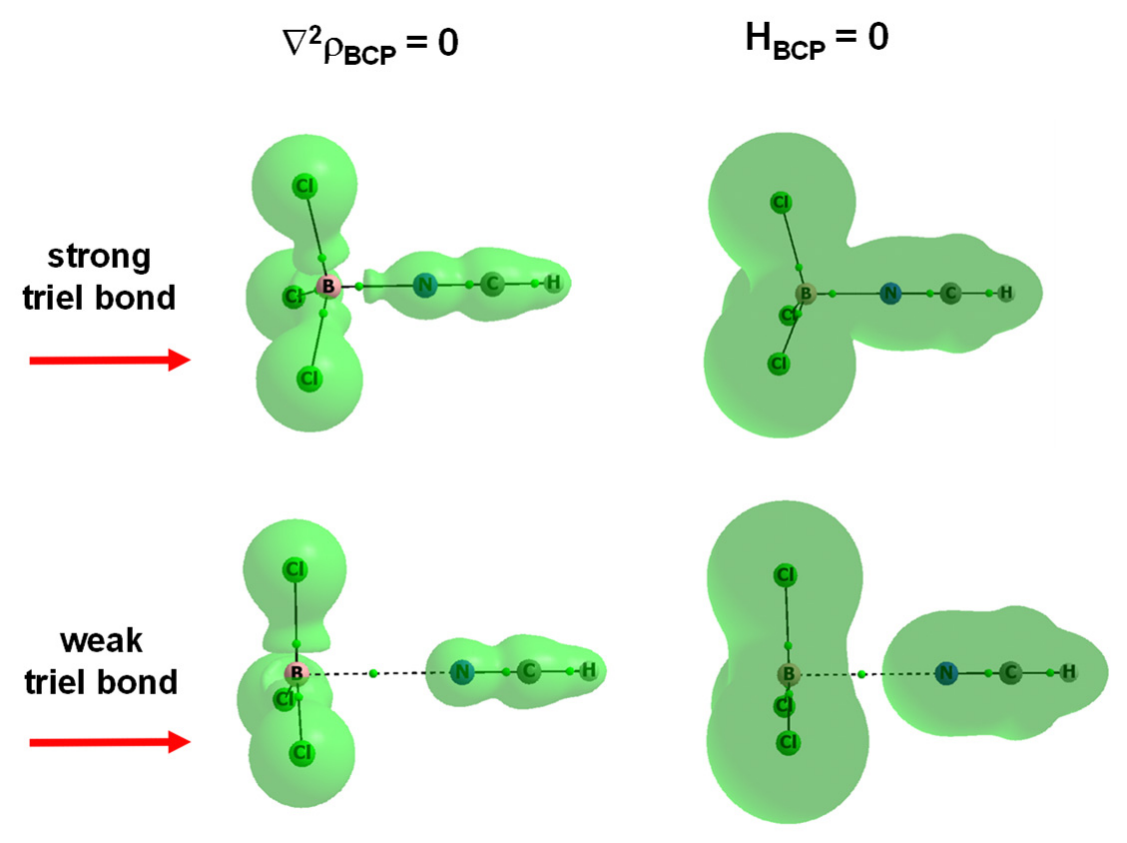
The reactive surfaces for two conformations of the Cl_3_B∙∙∙NCH complex, MP2/aug-cc-pVTZ results.

It is worth mentioning that in the case of weak or medium in strength interactions of the triel centres with Lewis bases, the trigonal planar configuration is only slightly deformed (if any), or such interactions lead to structures being intermediates between trigonal and tetrahedral ones. Figure 5 presents two conformations of the Cl_3_B∙∙∙NCH complex which correspond to energetic minima, they are characterised by the shorter and longer B∙∙∙N distance (i.e., to the strong triel bond and to the weak interaction, respectively). 

For example, for the weaker interaction the trigonal structure of BCl_3_ is only slightly deformed and for the strong one this deformation is directed towards the tetrahedral structure. The so-called reactive surfaces of two conformations of the Cl_3_B∙∙∙NCH complex are presented in Figure 5 that correspond to the zero values of the Laplacian of the electron density (∇^2^ρ) or of the total electron energy density (H). It is well known that the negative value of ∇^2^ρ at the bond critical point, BCP (∇^2^ρ_BCP_), corresponding to an interatomic link considered, indicates the covalent character of interaction [115,116]. However, if the ∇^2^ρ_BCP_ is positive the negative value of H at BCP (H_BCP_) indicates the partially covalent character of interaction [117,118,119,120]. The positive H_BCP_ value (and consequently positive ∇^2^ρ_BCP_) is attributed to weak and to medium strength interactions. One can see that for the Cl_3_B∙∙∙NCH configuration linked by the weak interaction, the B∙∙∙N BCP is located outside both surfaces of ∇^2^ρ and H of the zero value, therefore both ∇^2^ρ_BCP_ and H_BCP_ are positive. For the Cl_3_B∙∙∙NCH conformation linked by the stronger interaction, ∇^2^ρ_BCP_ is positive and H_BCP_ is negative as the reactive surfaces show (Figure 5).

**Figure 6:**
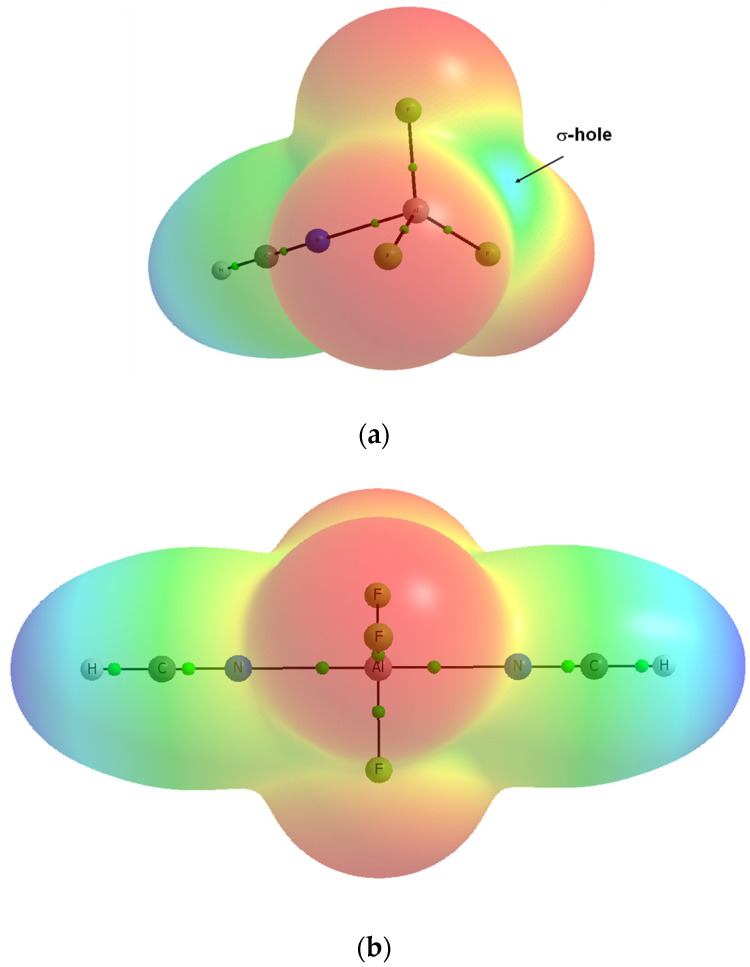
The EP surfaces of the electron density of 0.001 au for AlF_3_∙∙∙NCH (**a**) and AlF_3_∙∙∙(NCH)_2_ (**b**) species; from maximum positive EP (blue) to minimum negative EP (red); results correspond to the MP2/aug-cc-pVTZ level.

It was pointed out that the AlFH_3_^−^ tetrahedral structure possesses four σ-holes located in the extension of bonds to the aluminium centre. This structure and the similar triel tetrahedral structures may then further interact with the electron donating ligands that leads, similarly as in the charge assisted pnicogen bonds and tetrel bonds, to trigonal bipyramid structures. Figure 6a presents the molecular graph of the AlF_3_∙∙∙NCH structure which possesses approximately tetrahedral structure. One can see here the σ-hole located in extension of the N∙∙∙Al contact. The next interaction through the latter σ-hole leads to the trigonal bipyramid structure of AlF_3_∙∙∙(NCH)_2_ species. The surface of the electron density of 0.001 au with the EP map for the latter system is presented in Figure 6b. 

These examples of changes of structures that result from interactions show that the use of current names of interactions, at least for the majority of them, is not well fitted. For example, the triel bond may act through the σ-hole or through the π-hole. It was discussed in one of recent studies that for different elements of the 13 group the triel bonds possess different properties. For example, for the Al-centre they are mainly electrostatic in nature while for the Ga-centre they possess properties of covalent bonds [73].

On the other hand, different interactions lead to the same structural changes. The tetrel bonds, the charge assisted pnicogen bonds and the triel bonds of the tetradedral structures lead to the trigonal bipyramid conformation. This is why it seems reasonable to introduce another classification of interactions that is based on the structural changes of interacting species.

## 5. Changes of Structures of Interacting Centres Follow the VSEPR Model

It was justified in former sections that one may have numerous reservations to names of interactions and/or to their classifications that are often used in various studies. It seems that the simple way to mark any interaction is by giving names of centres being in a contact, for example, Br∙∙∙O, for the halogen bond, or H∙∙∙O for the hydrogen bond. In both cases, the special kinds of these interactions are indicated. In such a way the left side of this designation corresponds to the Lewis acid centre while the right side to the Lewis base. It is worth to note here that the two-centre definition of the hydrogen bond [38] was introduced recently, which is in line with the above-mentioned designations containing centres being in contact. However, these designations may be slightly extended if the information of the conformations of interacting centres is included. This is discussed in this section.

First of all, one can see that structural changes that follow interactions are in line with the VSEPR model. Briefly speaking, these are the following main assumptions of this model [102].

The arrangement of covalent bonds of the centre analyzed depends on the number of electron pairs in the valence shell; these are bonds, as well as nonbonding pairs as lone electron pairs.

The arrangement of valence electron pairs around the centre considered is to maximize their distances apart.

It is assumed that the non-valence electrons (inner electrons) with nucleus (i.e., the core) possess the spherical symmetry (or at least it is in force for the main groups elements).

The following structures are predicted by the VSEPR approach if the number of electron pairs increases from two to six: linear (L), trigonal (Tr), tetrahedral (Td), trigonal bipyramid (TBP), and octahedron (Oc) (Tr, Td, TBP, and Oc designations follow those applied by Martin [121]). They may be determined by the AX_n_E_m_ marks (according to ref. [102]) where A is the central atom, n is a number of X atoms linked by single bonds with A-centre, and m is a number of nonbonding or lone electron pairs. In other words, there are n+m electron pairs in the valence shell of the central atom. X atoms connected with A-centre are not necessarily the same.

It is not the aim of this study to discuss all of the assumptions of the VSEPR approach, the conclusions concerning the differences between the influence of nonbonding electron pairs and bonds on the structures mentioned above here are not also discussed. It is worth mentioning that a recent theoretical study on the Be^2+^(NCH)_n_ and Mg^2+^(NCH)_n_ (n up to six) species as well as on clusters containing both NCH ligands and CH_3_ groups [122], is well fitted to the VSEPR approach and to the σ-hole/π-hole concept.

**Figure 7:**
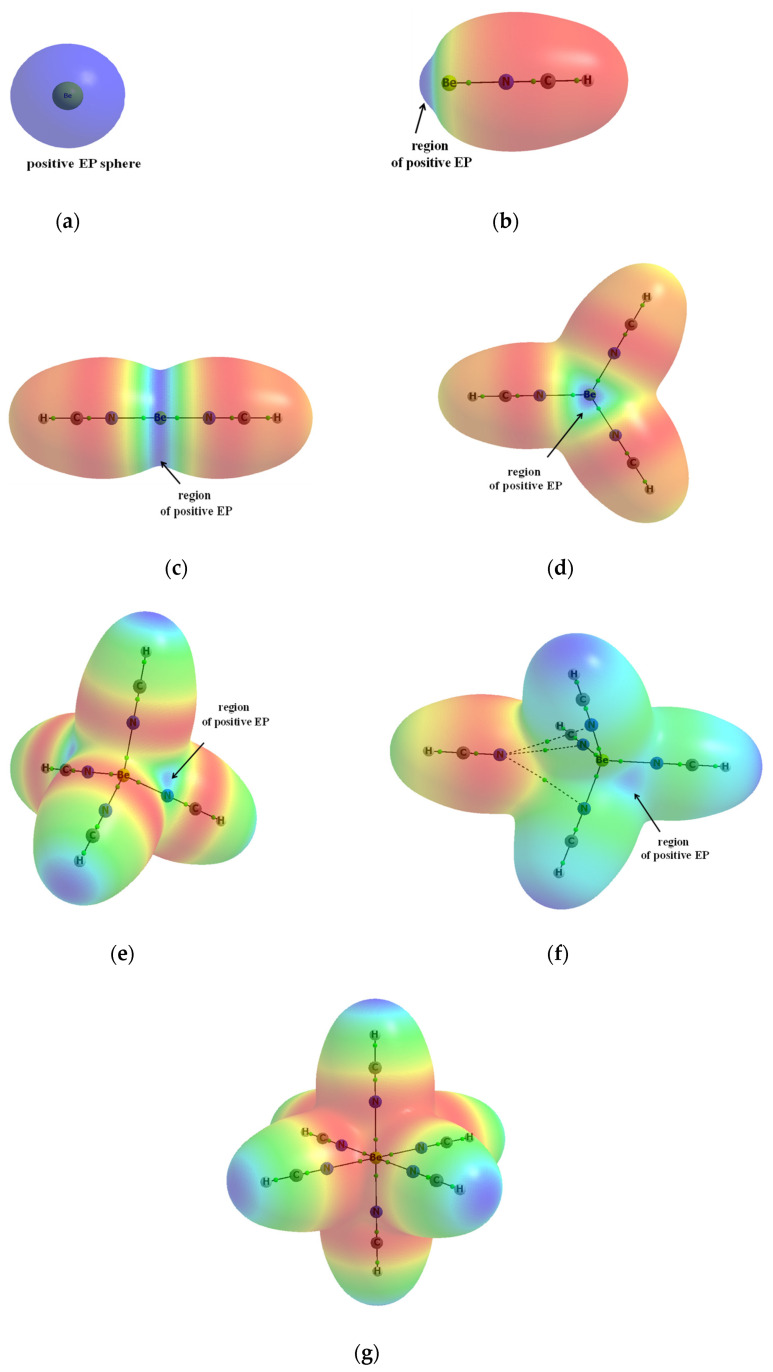
The EP surfaces of the 0.001 au electron density for the Be^2+^(NCH)_n_ species, Figure (**a**–**g**) for n = 0, 1…6, respectively These results correspond to the ωB97XD/aug-cc-pVTZ calculations.

Figure 7 shows molecular graphs with EP surfaces for the Be^2+^(NCH)_n_ clusters. The linear Be^2+^NCH and Be^2+^(NCH)_2_ systems are observed: the trigonal, tetrahedral and octahedral configurations exist for the Be^2+^(NCH)_3_, Be^2+^(NCH)_4_ and Be^2+^(NCH)_6_ clusters, respectively. The Be^2+^(NCH)_5_ cluster may be considered as formed from the Be^2+^(NCH)_4_ part deformed by the interaction with the next fifth NCH ligand. The whole Be^2+^(NCH)_5_ cluster is intermediate between the tetradedral structure with additional NCH species and the trigonal bipyramid structure that could contain five ligands.

It is worth mentioning that for the Mg^2+^(NCH)_5_ cluster, two configurations were found that correspond to the energetic minima. One of them is similar to the Be^2+^(NCH)_5_ system (Figure 7f) discussed here and the second one corresponds to the trigonal bipyramid [122]. For the other magnesium species (n = 1, 2, 3, 4, 6), a similar situation is observed as for the corresponding beryllium clusters.

Let us discuss the EP surfaces for the beryllium clusters. The Be^2+^ cation (Figure 7a) possesses the positive EP for the whole sphere. This positive EP sphere is modified, or one may say that it is restricted by interactions with the NCH ligands. The regions of positive EP in the Be^2+^(NCH)n clusters do not correspond to σ-holes or π-holes. For example, the σ-holes are related to covalent bonds (i.e., to the centre areas located at elongations of covalent bonds with this centre), while for the clusters analyzed the secondary bonds of Be^2+^ cation with ligands are observed.

For the Be^2+^NCH species (Figure 7b), the region of positive EP at beryllium centre is close in shape to the hemisphere. In the linear Be^2+^(NCH)_2_ cluster (Figure 7c) the ring of positive EP around Be-centre occurs. The Be^2+^(NCH)_3_ species (Figure 7d) possesses the positive EP at the Be-centre that could be classified as the π-hole if the criterion of the planarity of beryllium and its neighbouring nitrogen centres is taken into account. The Be^2+^(NCH)_4_ tetrahedral cluster (Figure 7e) contains four positive EP regions situated at the Be-centre in the elongation of the N∙∙∙Be links. These EP regions are similar to the σ-holes that occur in tetrel tetravalent elements. Similarly, in the Be^2+^(NCH)_5_ cluster (Figure 7f) the positive EP areas at beryllium centre occur. This structure may be considered as the Be^2+^(NCH)_4_ tetrahedron with four positive EP regions that is deformed by the next, fifth ligand which occupies one of the positive EP sites. Hence in the Be^2+^(NCH)_5_ cluster the regions of positive EP are restricted to three narrow sites. In the Be^2+^(NCH)_6_ octahedron (Figure 7g) there is no positive EP region for the beryllium centre.

**Figure 8:**
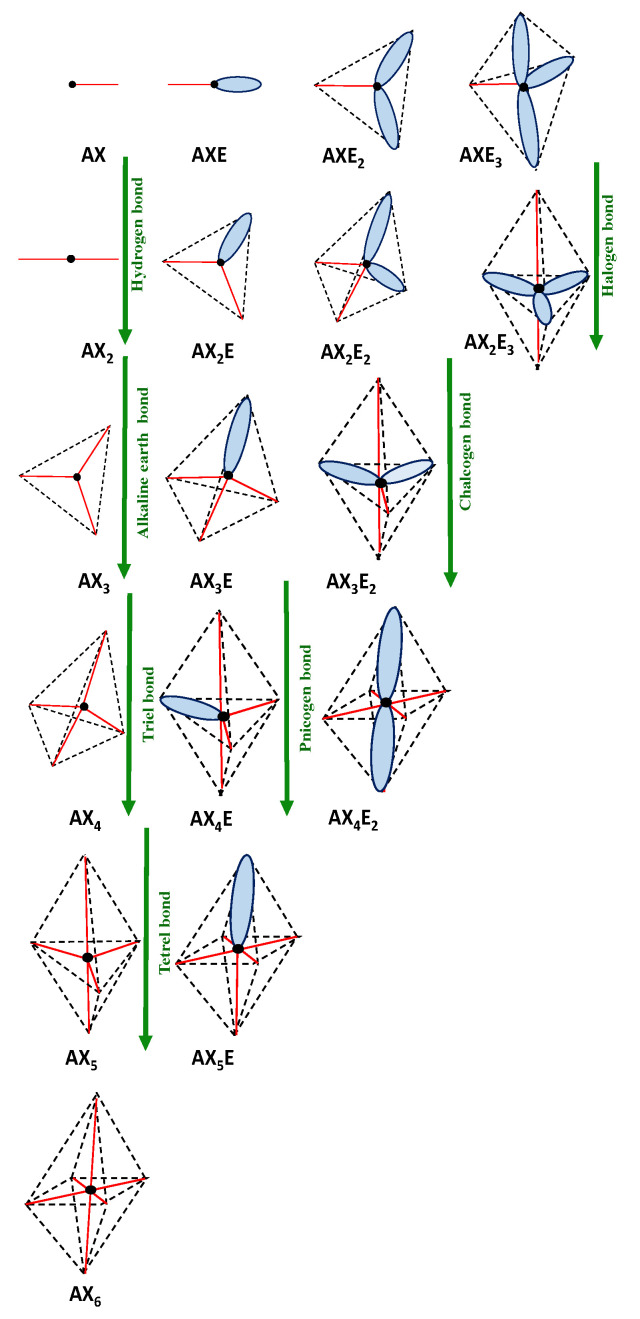
The VSEPR structures and their interrelations with interactions. The lone electron pairs presented as ellipsoids do not correspond to their shapes, they are introduced only to show schematically their localizations according to VSEPR model, the bonds are designated by red solid lines. The black circle corresponds to the centre considered, AX_n_E_m_ marks the type of structure where n and m subscripts correspond to the number of ligands (X) and electron pairs (E), respectively (n or m is omitted if it is equal to one). The green arrows show interactions that change one of structures into another one.

One may say that the configurations change if the subsequent ligands are included, from cation to the two-centre species containing one cation and one ligand, next to the linear system, the trigonal one, the tetrahedral, the trigonal bipyramid, and the octahedron. The inclusion of subsequent ligands is like the interaction of the positive EP beryllium region with the lone electron pairs of nitrogen centres. These Be∙∙∙N interactions may be treated similarly as the bonds in VSEPR approach. The first left column of Figure 8 shows schematically all of the beryllium configurations discussed here.

Figure 8, as a whole, is of more general meaning since it presents the configurations predicted by the VSEPR approach where not only bonds that influence the molecular shapes are taken into account but also lone electron pairs. The AX_n_E_m_ designations applied in this figure should be understood in the same way, as it was described earlier here. To simplify these designations, the differences between n ligands linked with A-centre are not specified.

**Scheme 3:**
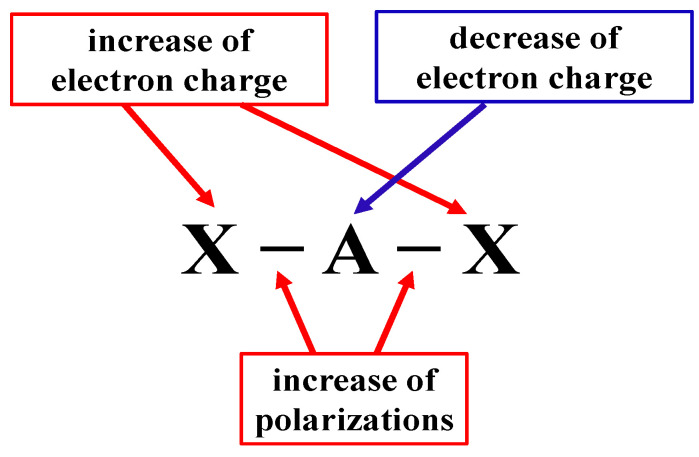
The electron charge shifts for the 3c/4e ω hypervalent bonds.

There are interesting interrelations between the positions of bonds and lone electron pairs. For example, in the trigonal bipyramid structure, TBP, the positions of vertices are not equivalent. This non-equivalency is discussed by Gillespie and Hargittai and its relation to positions of lone electron pairs and bonds was discussed [102]. However, it is also worth referring here to the ω 3c/4e hypervalent bonds discussed by Weinhold and Landis [101]. The latter concept explains correctly the locations of electron pairs. The lone electron pairs are usually characterised by electron occupancy equal to two or very close to this value. The bonds on the other hand, especially if they are strongly polarized, have occupancies sometimes much lower than two. This is why, for example, in the AX_3_E_2_ TBP structure the lone pairs occupy the positions in the triangle (see Figure 8). The lone electron pairs, neither one nor two, in TBP structures are directed to the axial vertices. Why? Because in such a case it is not possible to obey the octet rule. If the lone pairs are directed to vertices of the triangle thus the axial XAX arrangement may be considered as the ω 3c/4e hypervalent bond where both A-X links are strongly polarized, and where the shifts of the electron charge to the X-centres occur (see Scheme 3). The bond orders of the “partial” A-X bonds are lower than one (electron occupancies lower than 2). In such a way, the eight-electron valence shell of the A-centre is exceeded only slightly (if any). This is worth noting since three centres of the ω hypervalent bond have to be linear or nearly so [101]. Hence, it seems that following TBP structures contain such hypervalent bonds (the above mentioned AX_3_E_2_, as well as AX_2_E_3_, and AX_4_E). One can also expect the hypervalent bonds in the following octahedron (Oc) structures: AX_6_, AX_5_E, and AX_4_E_2_. The occurrence of the AX_3_E_3_ structure is less probable since in such a case only one hypervalent 3c/4e bond is possible and this does not prevent the excess of electrons above eight in the valence shell of the A-centre; it does not follow the octet rule.

Few interactions are involved in Figure 8. The hydrogen and halogen bonds are discussed in the next section. Let us look at other interactions. For example, the change of the tetrahedral (Td) AX_2_E_2_ configuration into the AX_3_E_2_ (TBP) may be realized through the chalcogen bond. There are numerous examples of such a situation. As the interaction of SFCl possessing the AX_2_E_2_ configuration with Cl^−^ anion that leads to the SFCl_2_^−^ anion. The latter is a very strong interaction, and thus the product of this reaction (interaction) is characterized by the AX_3_E_2_ configuration of the trigonal bipyramid where lone electron pairs are located in the plane of the triangle. However, it is more often observed that the chalcogen bond leads to the change of AX_2_E_2_ into the geometry being intermediate between Td (AX_2_E_2_) and TBP (AX_3_E_2_). The same concerns other interactions; the change of AX_4_ into AX_5_ (Td into TBP) may be realized only by the extremely strong tetrel bond where the trigonal bipyramid structure corresponds to the transition state of the S_N_2 reaction [63]. The ClCH_3_∙∙∙∙F^−^ tetrel interaction is an example that is the preliminary stage of the S_N_2 reaction. The AX_4_ configuration of the ClCH_3_ species changes into the AX_5_ configuration of ClCH_3_F- transition state and next for products, Cl^−^∙∙∙∙CH_3_F, the CH_3_F possesses the AX_4_ configuration. 

It is worth mentioning that not only the tetrel bond may realize the AX_4_ → AX_5_ change. It may be also the charge assisted pnicogen bond described in the former section. The same concerns other interactions indicated in Figure 8. Each of changes shown in this figure is realized by various types of interactions in various types of structures. The following changes of structures were shown for the interactions usually characterized as the σ-hole bonds; halogen, chalcogen, pnicogen, and tetrel bonds; AXE_3_ → AX_2_E_3_, AX_2_E_2_ → AX_3_E_2_, AX_3_E → AX_4_E and AX_4_ → AX_5_, respectively. The AX_3_ → AX_4_ change may be realized by the triel bond while the AX_2_ → AX_3_ by the beryllium bond or other alkaline earth bonds. However, as it was noted above here, each of these changes of conformations may be realized by at least few different interactions (marked by hitherto applied names in various studies). Similarly, each of interactions may realize various changes of conformations. For example, the halogen bond if concerns the multivalent halogen centre may concern different changes of structures from thet change mentioned earlier here (i.e., AXE_3_ → AX_2_E_3_) typical for the monovalent halogens [123]).

It is also worth mentioning that conformations presented in Figure 8 often do not correspond to real electronic structures. However, their changes described earlier here and particularly their interactions are well fitted into the structures predicted by the VSEPR approach (Figure 8). The structure of water is an example since the sp^3^ hybridization model is often presented here with the oxygen located in the centre of the tetrahedron and both two OH bonds and two lone electron pairs directed towards the vertices. Numerous structural properties of water clusters as well as numerous crystal structures containing water molecules are in line with this model. It is not the aim of this study to discuss it in detail. It is worth noting, however, that this is not true picture of the water molecule; two lone electron pairs are not equivalent and the sp^3^ hybridization model is not correct [124].

The idea of the classification of interactions proposed here is used to simplify the situation by giving the symbols of atoms being in contact and to indicate the change of configurations. The latter information concerns the Lewis acid centre that is usually included hitherto in names of interactions. Names related to the Lewis base centres are rather rare. It seems that it is sufficient to present only two centres being in contact, Lewis acid one at the left site and the Lewis base centre at the right site. For example, the mark S∙∙∙O is much more informative than the name chalcogen bond. The change of conformations of the Lewis acid centre may be also included, namely as the designation (AX_2_E_2_ → AX_3_E_2_) S∙∙∙O contains a lot of information and it is very simple. It informs that the Lewis acid centre containing two bonds and two lone electron pairs (Td, tetrahedron structure) is in a contact with the oxygen Lewis base centre. This interaction is directed to the change of the sulphur coordination into one that contains three bonds to the sulphur, and two electron pairs, (TBP, trigonal bipyramid). The designations of interactions proposed here may be slightly expanded if the A-centre being in contact with the Lewis base is monovalent. This is described in the next section.

## 6. The Hydrogen Bond and the Halogen Bond Related to VSEPR Model

The question remains how we should classify hydrogen bond interactions. The hydrogen atom which possesses one electron is monovalent and its configuration may be attributed to AX two-atoms system where an H-atom of the positive charge is linked with another atom, most often characterized by high electronegativity. The H-atom interacting with the electron donor tries to achieve the AX_2_ linear configuration with the proton located in the mid-point of the X∙∙∙X distance. However, such a situation is observed only for very strong hydrogen bonds, such as for the [FHF]^−^ anion or for the O∙∙∙H∙∙∙O link in the H_2_O∙∙∙H_3_O^+^ complex where the proton is very close to the O∙∙∙O mid-point [125]. In latter cases one may say of the hypervalent hydrogen centres [28] or of the 3c/4e hypervalent ω-bonds [101]. In the case of the above-mentioned hydrogen bonds, the following designations may be applied: (AX → AX_2_) FH∙∙∙F and (AX → AX_2_) OH∙∙∙O. These are slightly extended designations in comparison with those proposed in the former section since the centres connected with hydrogen are indicated. This is in line with the former, commonly applied in the literature designations where three atoms forming the hydrogen bonded bridge are usually indicated. The same convention may be applied for all Lewis acid centres involved in interactions that are monovalent. The hydrogen centre involved in the hydrogen bond interaction may further interact with the Lewis base ligand. This is the bifurcated hydrogen bond and the configuration of the hydrogen centre may be marked as AX_3_. One may say that two systems, A-H∙∙∙B and A-H∙∙∙B’, occur here. It was found in an early study that the sum of AHB, AHB´, and BHB´ angles amounts approximately 360° that confirms the assumption of the AX_3_ structure [126,127]. The greater number of electrons donating ligands that coordinate H^+^ or H^−^ ions was discussed [128]. However it seems to be less probable because of the small size of the hydrogen ion.

There is an issue related to interactions with π or σ-electrons that act as the Lewis base sites. One may refer to the A-H∙∙∙π and A-H∙∙∙σ types of hydrogen bonds [38]. For example, for the T-shaped acetylene dimer and the T-shaped complex of hydrogen fluoride with molecular hydrogen the following designations may be applied: (AX → AX_2_) CH∙∙∙π and (AX → AX_2_) FH∙∙∙σ. The similar designations may be used for other interactions, apart from hydrogen bonds, with the π or σ-electrons acting as nucleophiles.

This is difficult to classify for any conformation wherein the monovalent halogen centre is interacting with the Lewis base. However, one may refer to idealized situation that this centre not involved in any interaction possesses the AXE_3_ configuration, in such a way one may indicate the following designation for the interaction in the dimer of p-iodobenzonitrile [129] (for example, (AXE_3_ → AX_2_E_3_) CI∙∙∙N). The different situation is observed for the multivalent halogens possessing characteristics of Lewis acid centres. The interactions of trivalent and pentavalent bromine species with the NCH and N_2_ ligands as well as with the fluorine, chlorine, and bromine anions have been discussed, as well as changes of the conformations of halogen centres resulting from interactions [123]. For example, for the BrF_3_∙∙∙Cl^−^ and BrF_5_∙∙∙F^−^ complexes, the following designations may be applied: (AX_3_E_2_ → AX_4_E_2_) Br∙∙∙Cl and (AX_5_E → AX_6_E) Br∙∙∙F, respectively. One may mention here the study on crystal structures containing multivalent halogens (bromine and iodine centres) that interact with electron donors [130]. The latter interactions lead to changes of configurations which are in line with the VSEPR model. The analysis of interactions in crystals which are in line with the concepts presented in this study is in progress.

The AX_6_E configuration corresponding to the (AX_5_E → AX_6_E) Br∙∙∙F interaction mentioned above is not even included in Figure 8. Thus, one can see that the scheme presented there may be extended on other configurations, and that other interactions may be inserted there since only few examples are presented in this figure. One example of the protonation of the water molecule may be mentioned, H_2_O + H^+^ → H_3_O^+^ + H^+^→ H_4_O^2+^ [131], which corresponds to the following changes of conformations: AX_2_E_2_ → AX_3_E → AX_4_. The latter example concerns the change of the conformation of the Lewis base centre (oxygen) while the whole classification related to the VSEPR approach proposed here is based on changes of conformations of interacting Lewis acid centres. One can see from the example of protonation presented above that the classification may be extended on changes of the Lewis base centres. The designations of interactions may be also extended to include more information about Lewis base centres being in a contact with electron acceptors. However, the classification and designations of interactions proposed here seem to be a good compromise between the information included and the simplicity. Few examples of commonly known interactions at the end of this section may be presented: the water dimer is linked by the (AX → AX_2_) OH∙∙∙O interaction, the interaction in the ammonia-borane dimer, known in various studies as dihydrogen bond is designated as (AX → AX_2_) NH∙∙∙H or the interaction in imidazole-BeH_2_ complex as (AX_2_ → AX_3_) Be∙∙∙N.

## 7. Conclusions

The names and classifications of interactions that are applied so far in various studies are discussed here. These interactions, particularly strong ones, often lead to changes of configurations of the units that are in contact. The following configurations that are predicted by the Valence-shell Electron-pair Repulsion model, VSEPR, occur very often; linear (L), trigonal (Tr), tetrahedral (Td), trigonal bipyramid (TBP), and octahedral (Oc). The change of one of these structures into another one may be realized by different interactions. For example, the change of Td configuration into TBP occurs in a case of strong tetrel bonds and strong charge assisted pnicogen bonds. Similarly, for the same name of an interaction, various structural changes may be observed. There are numerous other reservations related to the commonly applied names and classifications of interactions.

Hence the simple designations of interactions were proposed here that inform of sites being in a contact and of the change of configuration of the Lewis acid centre. It seems that these new designations are much more informative than those applied before in numerous studies. Additionally, these new names are in agreement with mechanisms that accompany the formation of various Lewis acid–Lewis base interactions. For all of them, the electron charge shift from the Lewis base unit into the Lewis acid one is observed and other shifts of charges occur within these units that allow them to protect the doublet/octet electron structures of their centres which are in contact.

## Data Availability

Not applicable.
